# Serial Casting for Early-Onset Scoliosis

**DOI:** 10.3390/jcm14124329

**Published:** 2025-06-18

**Authors:** Jakub Adamczyk, Slawomir Duda, Wojciech Kacki, Barbara Jasiewicz, Tomasz Potaczek

**Affiliations:** Department of Orthopedics and Rehabilitation, Medical College, Jagiellonian University, 34-500 Zakopane, Poland; j.adamczyk@uj.edu.pl (J.A.); slawomir.duda@uj.edu.pl (S.D.); wojciech.kacki@uj.edu.pl (W.K.); tomasz.potaczek@uj.edu.pl (T.P.)

**Keywords:** scoliosis, casts, spine/growth and development, thorax/growth and development, orthotic devices, child, preschool, infant

## Abstract

**Background**: Early-onset scoliosis (EOS) is a severe spinal deformity that can compromise thoracic development and pulmonary function if left untreated. While Mehta casting is widely used to manage deformity non-surgically in young children, its effects on spinal and thoracic growth remain underexplored. **Methods**: In this retrospective case series, 15 children with EOS underwent serial elongation–derotation–flexion (EDF) Mehta casting. Radiographic assessments were performed pre-treatment, post-casting, and at follow-up, including measurements of Cobb angle, rib–vertebral angle difference (RVAD), Th1–Th12 spinal length, coronal chest width (CCW), and space available for lung (SAL). Growth rates were estimated based on the duration of treatment. Correlation analyses were conducted to examine associations between baseline deformity and structural outcomes. **Results**: Serial casting reduced the mean Cobb angle by 22.2° and RVAD by 15.5°. During treatment, measurable increases were observed in Th1–Th12 length (mean: 2.93 cm), CCW (1.12 cm), SAL-L (2.60 cm), and SAL-R (2.98 cm). Estimated annual growth was significantly greater in children with lower initial Cobb and RVAD values. In contrast, total casting duration showed no consistent correlation with growth outcomes. **Conclusions**: Mehta casting is effective not only in correcting spinal deformity but also in supporting thoracic and axial growth in children with EOS. Early application in flexible, less severe curves may optimize structural outcomes and preserve thoracic development during early growth.

## 1. Introduction

Early-onset scoliosis (EOS) is defined as a spinal deformity presenting before the age of ten. Spinal deformities in this population may arise from various underlying causes, including congenital vertebral anomalies, neuromuscular dysfunctions such as cerebral palsy or spinal muscular atrophy, skeletal dysplasias, inherited syndromic conditions like Marfan syndrome or Ehlers–Danlos syndrome, or remain idiopathic without an identifiable cause [1].

The natural history of EOS underscores the critical importance of early intervention. The so-called “Golden Period” refers to the critical window from birth to approximately 8 years of age, during which the thoracic cage undergoes rapid expansion and the lungs experience exponential alveolar multiplication. Disruption of spinal alignment during this stage may compromise thoracic volume and impede pulmonary development, leading to long-term functional impairment. The primary therapeutic aim during this period is not only curve control, but also preservation of thoracic growth potential to avoid thoracic insufficiency syndrome [2]. Structural progression beyond a 70° Cobb angle, particularly before age six, is strongly correlated with irreversible pulmonary compromise [3,4]. Thus, timely treatment during the “Golden Period” of thoracic and spinal growth is essential.

Modern management strategies emphasize not only deformity control but also the preservation of spinal growth, thoracic volume, and pulmonary function [5]. Growth-accommodating treatments, such as serial corrective casting, aim to leverage natural growth as a corrective force [6,7]. Among these, Mehta casting—based on elongation, derotation, and flexion (EDF) principles—has emerged as a first-line therapy, particularly for young children with idiopathic or flexible curves [8]. Early reports demonstrated that Mehta casting can significantly reduce Cobb angles and, in some cases, delay or even prevent the need for surgical intervention [9,10,11,12].

Despite its widespread clinical use, relatively few studies have examined the impact of Mehta casting on spinal growth itself, particularly using longitudinal radiographic measures of axial elongation. Most existing literature has focused primarily on curve magnitude and rotational parameters. However, because the overarching aim of EOS treatment is not just deformity control but preservation of spinal and thoracic growth, further investigation is warranted into how casting affects structural development over time.

We therefore conducted a retrospective case series with the following objectives:

The primary objective was to quantify changes in spinal length (Th1–Th12), coronal chest width (CCW), space available for lung (SAL) and coronal balance after serial Mehta casting in children with EOS.

Secondary objectives were (1) to assess correlations between baseline deformity and subsequent structural growth, and (2) to explore the role of early conservative management in preserving thoracic development.

By focusing on both deformity correction and thoracic growth outcomes, this study seeks to offer a more comprehensive understanding of the biomechanical and developmental effects of Mehta casting.

## 2. Materials and Methods

### 2.1. Study Design and Participants

This was a retrospective case series conducted between 2017 and 2024 at a tertiary pediatric orthopedic center. The study population included children diagnosed with early-onset scoliosis (EOS), defined as a spinal curvature developing before the age of ten. Patients were eligible for inclusion if they demonstrated progressive scoliosis with a Cobb angle ≥30°, a rib–vertebral angle difference (RVAD) >20°, and/or a phase 2 rib–vertebra relationship, indicating overlap of the ribs and vertebrae at the apex of the curve.

Children were excluded if they had a prior history of spinal surgery, had undergone bracing before casting, or had incomplete radiographic records at follow-up.

### 2.2. Intervention: Serial Mehta Casting Protocol

**Figure 1:**
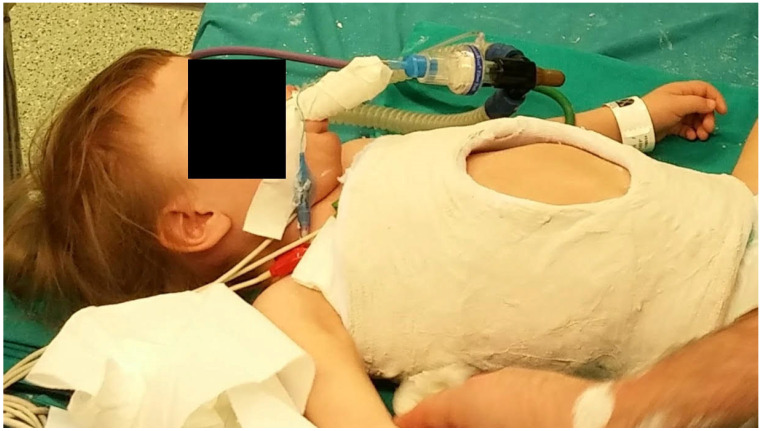
A child just after applying a cast on a frame. Still under general anesthesia. The last part of the procedure is cast trimming.

All included patients underwent serial casting using the elongation–derotation–flexion (EDF) casting technique, which is performed under general anesthesia. The procedure involves applying gentle longitudinal traction (elongation) to reduce curve magnitude, derotation of the torso to restore normal vertebral alignment, and controlled flexion to bring the spine into a more balanced position. The cast is applied on a specialized frame (e.g., Risser or Mehta frame), allowing for three-dimensional correction while preserving thoracic expansion (Figure 1). Casts extended from the axilla to the pelvis and were applied every 8 to 12 weeks, depending on the child’s growth and clinical progress. The number of casts per patient and duration of treatment were recorded.

### 2.3. Radiographic Measurements

Radiographic assessments were performed at three standardized time points: (1) prior to the first cast (pre-treatment), (2) following the final cast (post-treatment), and (3) during follow-up, conducted at least six months post-treatment.

**Figure 2:**
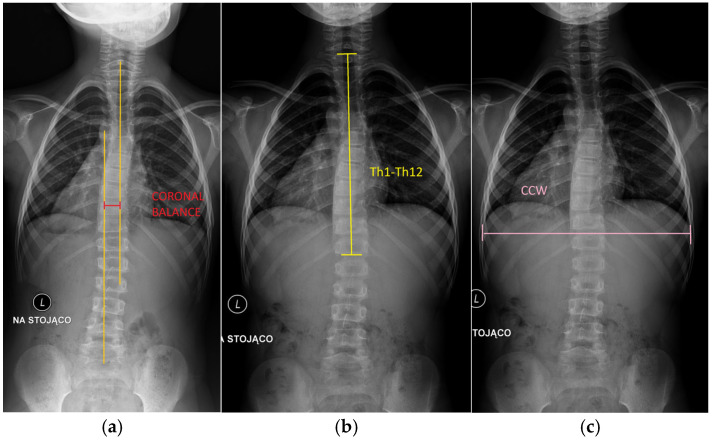
Measurements used in the manuscript: (**a**) coronal balance; (**b**) Th1–Th12 spinal length (the distance between first thoracic vertebra and twelve thoracic vertebra); (**c**) coronal chest width.

**Figure 3:**
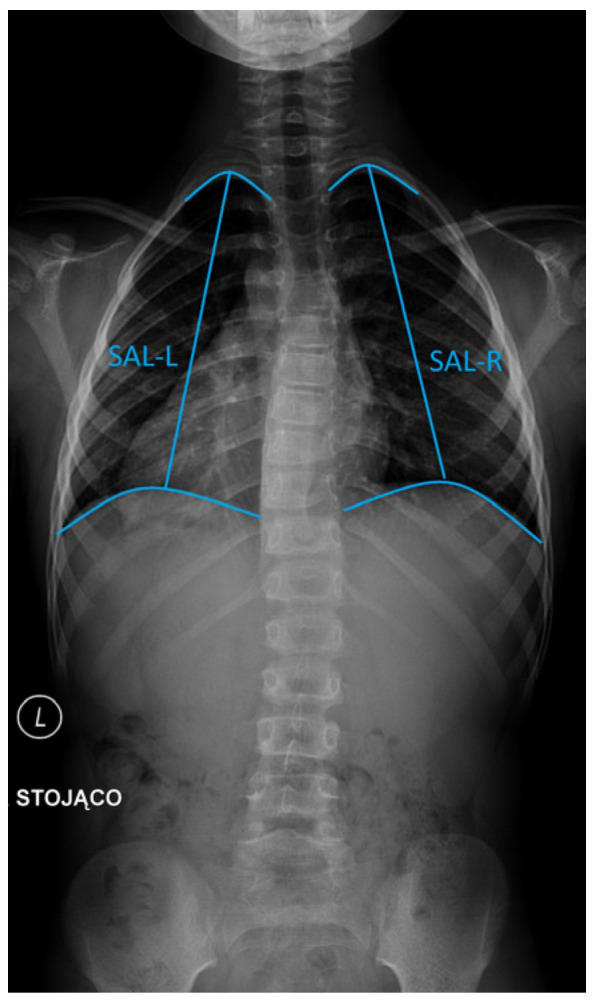
Measurements used in the manuscript: space available for lung (SAL) on the left (SAL-L) and right (SAL-R) hemithorax.

The following parameters were measured from standing posteroanterior spine radiographs (with use of Carestream v.12.2.1.0104 software):

- Cobb angle (°): Measured as the angle between the upper endplate of the most tilted vertebra above the curve apex and the lower endplate of the most tilted vertebra below the apex, following standard protocols.
- RVAD (rib–vertebra angle difference (°): Calculated at the apex of the curve as the difference between the angles formed by each rib head and the corresponding vertebral body on each side.
- Coronal balance (cm): Defined as the horizontal distance from the C7 plumb line to the central sacral vertical line (CSVL) on posteroanterior radiographs. A positive value indicates a shift to the right, and a negative value indicates a shift to the left. This parameter reflects overall spinal alignment in the coronal plane (Figure 2).

- Th1–Th12 spinal length (cm): Defined as the vertical linear distance from the superior endplate of Th1 to the inferior endplate of Th12 on posteroanterior radiographs, representing thoracic spinal height.
- Coronal chest width (CCW) (cm): Measured on posteroanterior radiographs as the maximal horizontal distance between the medial cortices of the ribs at the level of T8, providing an estimate of transverse thoracic development.
- Space available for lung (SAL) on the left (SAL-L) and right (SAL-R) hemithorax (cm): Determined separately for each hemithorax as the vertical distance from the midpoint of the most cephalad rib to the center of the ipsilateral hemidiaphragm [13] (Figure 3).

Spinal and thoracic growth were quantified by comparing measurements between post-treatment and follow-up time points (e.g., ΔTh1–Th12 = follow-up–post-treatment). All radiographic measurements were performed using digital imaging software by two independent observers. Discrepancies exceeding 5% were observed in 4 out of 90 total measurements (4.4%) and were resolved through consensus discussion between the readers.

### 2.4. Statistical Analysis

Statistical analyses were performed using IBM SPSS Statistics version 29 (IBM Corp., Armonk, NY, USA). Descriptive statistics were used to report demographic and baseline variables. The Shapiro–Wilk test was used to evaluate the normality of data distribution. For longitudinal comparisons of radiographic parameters across time points, repeated measures ANOVA was used for normally distributed data. Correlation analyses between baseline variables (e.g., Cobb, RVAD) and growth outcomes (e.g., ΔTh1–Th12, ΔSAL) were performed using Pearson’s or Spearman’s correlation coefficients as appropriate. A *p*-value of <0.05 was considered statistically significant.

### 2.5. Ethical Approval

This study was approved by the Institutional Review Board (IRB No 118.0043.1.23.2024) in accordance with the Declaration of Helsinki. Written informed consent was obtained from the parents or legal guardians of all participants prior to enrolment.

## 3. Results

### 3.1. Study Population

**Figure 4:**
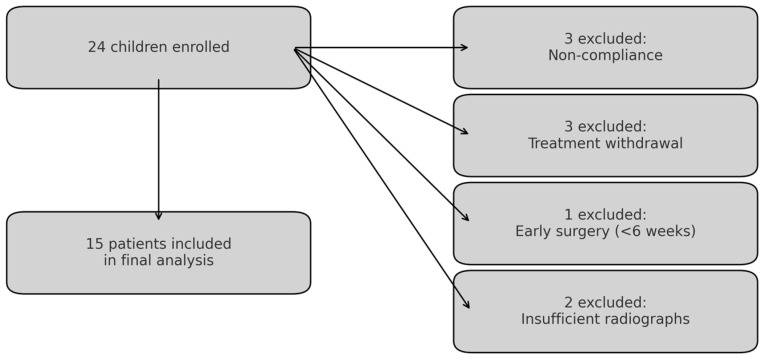
Flow diagram of patient enrollment, exclusion, and analysis.

Of the 24 children initially enrolled, 9 were excluded for reasons shown in Figure 4, resulting in a final analysis cohort of 15 patients.

**Figure 5:**
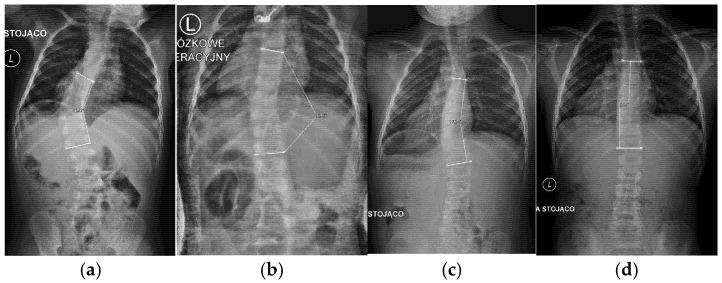
Patient GM, with early onset scoliosis: (**a**) before treatment, age 1.5 years, Cobb angle 43°; (**b**) during casting, age 2 years; (**c**) after treatment, age 4.1, Cobb angle 17°; (**d**) follow up, age 7.11 years, Cobb angle 3°.

The final cohort of 15 children with EOS was analyzed. The mean age at the beginning of Mehta casting was 2.14 years (Figure 5). Each patient underwent, on average, 5.0 serial casts, with a mean casting interval of 10.9 weeks.

### 3.2. Radiographic Deformity Correction

At follow-up, substantial improvements in radiographic deformity were observed following serial Mehta casting.

The mean Cobb angle decreased from 49.5° (SD = 12.84°, range 26.0–78.0°) at baseline to 27.3° (SD = 21.69°, range 8.0–68.0°) at follow-up, representing an average reduction of 22.2°.

Similarly, the rib–vertebra angle difference (RVAD) improved from 29.5° (SD = 11.64°, range 15.0–50.0°) to 14.0° (SD = 13.49°, range 0.0–47.0°), with a mean reduction of 15.5°.

These changes demonstrate effective deformity correction in both the coronal and rotational planes.

Detailed numeric values for each radiographic parameter, including minimum and maximum ranges, are presented in Table 1.

### 3.3. Spinal and Thoracic Growth

Between the end of the casting period and the follow-up, measurable increases were observed in all structural dimensions assessed.

The mean Th1–Th12 spinal length increased from 16.50 cm (post-treatment) to 19.43 cm at follow-up, representing an average elongation of 2.93 cm (SD = 1.46 cm, range −0.5 to 8.4 cm).

Coronal chest width (CCW) increased by a mean of 1.12 cm (SD = 1.37 cm, range 0.1 to 2.9 cm), from 17.03 cm to 18.15 cm.

The space available for lung (SAL) also expanded. On the left, SAL increased from 12.50 cm to 15.10 cm (mean Δ = 2.60 cm, SD = 1.42 cm), and on the right, from 11.85 cm to 14.83 cm (mean Δ = 2.98 cm, SD = 1.35 cm).

These results indicate that serial Mehta casting was associated not only with spinal elongation but also with thoracic expansion, bilaterally.

### 3.4. Estimated Annual Growth Rates

To better account for variation in treatment durations among patients, we estimated the annualized growth rates of spinal and thoracic parameters by dividing the absolute change in each dimension by the corresponding treatment duration (in years).

The mean annual growth in Th1–Th12 spinal length was 2.71 cm/year (SD = 1.33 cm/year, range −0.46 to 12.60 cm/year).

For coronal chest width (CCW), the mean annual increase was 0.96 cm/year (SD = 1.07 cm/year, range 0.09 to 4.35 cm/year).

SAL-L increased at a rate of 2.23 cm/year (SD = 1.26 cm/year, range −0.94 to 7.65 cm/year), while SAL-R increased by 2.64 cm/year (SD = 1.29 cm/year, range −0.60 to 9.45 cm/year).

These annualized metrics provide insight into the relative pace of axial and thoracic development during casting, adjusted for individual variation in treatment timelines.

### 3.5. Predictors of Annual Growth

We explored whether baseline deformity severity was associated with structural growth during the casting period.

Cobb angle at baseline negatively correlated with Th1–Th12 spinal growth (r = −0.60), coronal chest width (r = −0.48), SAL-L (r = −0.49), and SAL-R (r = −0.51), indicating that children with less severe curves showed greater structural improvement.

RVAD at baseline showed similar trends: correlations with Th1–Th12 growth (r = −0.40), SAL-L (r = −0.34), and SAL-R (r = −0.34).

These findings suggest that the initial magnitude of deformity is a meaningful predictor of both axial and thoracic growth potential in patients undergoing Mehta casting.

### 3.6. Treatment Duration Analysis

To determine whether the total duration of casting treatment influenced structural growth, we assessed correlations between treatment length and changes in Th1–Th12 spinal length, CCW, and SAL.

The analysis revealed no strong or consistent associations. Specifically, the correlation between total treatment duration and Th1–Th12 growth was weakly negative (r = −0.18), and the correlations with CCW (r = 0.12), SAL-L (r = 0.07), and SAL-R (r = −0.08) were negligible.

These findings suggest that longer treatment duration alone does not strongly predict structural growth, underscoring the importance of baseline deformity and casting quality rather than time-based metrics.

## 4. Discussion

Early-onset scoliosis (EOS) presents a significant clinical challenge due to its association with progressive spinal deformity, impaired thoracic development, and long-term respiratory compromise if left untreated [1,2]. Timely intervention during this critical period of growth is essential to prevent thoracic insufficiency and preserve pulmonary function [3,4]. In this prospective study, we demonstrate that serial Mehta casting in children with EOS not only achieves significant deformity correction but also supports meaningful spinal and thoracic growth. The mean Cobb angle was reduced by over 22°, and RVAD by approximately 16°, reflecting substantial improvement in both coronal and rotational alignment. Importantly, longitudinal radiographic assessment revealed measurable increases in Th1–Th12 spinal length, coronal chest width (CCW), and bilateral space available for lung (SAL). When adjusted for treatment duration, the estimated annual growth in spinal and thoracic dimensions confirmed continued axial and chest development during the casting period. Moreover, initial deformity severity—particularly Cobb angle and RVAD—was a stronger predictor of structural outcomes than treatment duration, reinforcing the importance of early and targeted intervention.

Several prior studies have highlighted the capacity of Mehta casting to achieve curve correction in early-onset scoliosis, particularly when initiated before the age of three [3,6]. Mehta herself reported that spinal deformity could be reversed in up to 94% of children treated early with serial elongation–derotation–flexion (EDF) casts, particularly in idiopathic and flexible curves [8]. More recent analyses have also demonstrated substantial Cobb angle reductions and curve stabilization across various EOS etiologies [11,14]. However, most of these studies have focused on coronal plane correction and clinical outcomes, while relatively few have assessed the impact of casting on longitudinal growth of the spine and thorax. Demirkiran et al. [15] showed a mean annual growth of Th1–Th12 spinal height of 0.81 cm (range, 0.62 to 1.06 cm) per year, despite the presence of congenital anomalies. Cao et al. [16] compared the annual growth rate between patients with and without spinal congenital anomalies, concluding that in the non-congenital group, the pace was significantly higher. Li et al. [17] on the other hand presented an increase both in Th1–Th12 length and coronal chest width. Our findings expand this body of evidence by providing radiographic estimates of annual growth in Th1–Th12 spinal height, coronal chest width, and space available for lung (SAL). In doing so, we offer additional support for the hypothesis that casting can preserve and even enhance axial and thoracic growth during critical developmental windows.

The findings of this study carry important clinical implications. First, the observed correlation between lower initial Cobb angles and RVAD values with greater spinal and thoracic growth suggests that early and less severe deformities are more responsive to non-surgical correction and structural remodeling. This reinforces the concept that Mehta casting is most effective when applied early in the disease course and in patients with flexible, idiopathic curves [8,10]. Additionally, our results challenge the assumption that prolonged bracing duration necessarily correlates with better outcomes. Instead, treatment efficacy appears to be more closely related to initial curve severity and the dynamic correction achieved during each cast interval [10,11]. These insights support a more individualized treatment approach, where careful radiographic assessment at baseline may inform prognosis and guide shared decision-making with families. Furthermore, the preservation of thoracic development during casting underscores the dual therapeutic role of this technique in both curve management and pulmonary protection—particularly relevant in a patient population at risk for thoracic insufficiency syndrome [18,19].

This study has several strengths. It is one of the few prospective investigations to provide a radiographically grounded, quantitative assessment of both spinal and thoracic growth in patients undergoing Mehta casting for EOS. Unlike previous studies that have focused predominantly on Cobb angle correction, this work integrates axial and thoracic dimensions—Th1–Th12 length, CCW, and SAL—offering a more holistic view of patient development. The use of estimated annual growth rates adds an important temporal dimension to growth interpretation, and the correlation of these parameters with initial deformity severity enhances its clinical relevance. Together, these elements provide a more granular and physiologically meaningful understanding of treatment outcomes and support evidence-based refinements in patient management. However, certain limitations should be acknowledged. A notable limitation is the exclusion of 9 out of 24 initially enrolled patients (37.5%), which may introduce selection bias. These exclusions were due to non-compliance, withdrawal from treatment, early surgical conversion, or incomplete radiographic data. While such criteria were applied consistently, they may have favored patients who were more adherent, clinically stable, or had more complete follow-up, potentially influencing the generalizability of the results. The small sample size reflects the rarity of EOS and the strict inclusion criteria applied. Nonetheless, key outcome measures demonstrated statistically significant changes, supporting the robustness of the findings despite the limited sample. The absence of a non-casted control group restricts direct comparison with other treatment modalities. Moreover, while the use of standardized radiographic intervals improves internal consistency, variations in follow-up duration and individual growth trajectories may introduce measurement variability. Lastly, although SAL serves as a proxy for thoracic volume, the lack of direct pulmonary function testing prevents correlation of structural changes with respiratory outcomes.

Future studies should aim to validate these findings in larger, multicenter cohorts with longer follow-up to assess the durability of both deformity correction and structural growth. Comparative studies between Mehta casting and early surgical interventions—such as growth-friendly instrumentation, which refers to expandable systems like traditional growing rods [20] or magnetically controlled growing rods [21] designed to accommodate spinal growth—could help clarify optimal treatment pathways for different EOS subtypes. In this context, recent advances in additive manufacturing, including the use of 3D-printed, patient-specific implants, may further improve outcomes in anatomically complex cases [22]. Incorporating pulmonary function assessments would also allow a more comprehensive evaluation of thoracic volume expansion and its correlation with respiratory outcomes. Additionally, identifying radiographic or clinical predictors of poor response to casting could refine patient selection criteria and improve treatment algorithms. Ultimately, continued investigation is essential to fully understand the biomechanical and physiological benefits of early conservative management in EOS.

## 5. Conclusions

This study suggests that Mehta casting may support both curve correction and spinal/thoracic growth in children with early-onset scoliosis, particularly when applied early to flexible curves. Based on these findings, we recommend the following clinical actions:

1. Early referral of children with EOS to specialized centers during the “golden period” of thoracic growth.
2. Assessment of curve flexibility to determine candidacy for non-surgical correction.
3. Implementation of structured follow-up protocols, including growth monitoring and radiographic assessment.

These recommendations may help optimize spinal and thoracic outcomes prior to considering surgical intervention. Further controlled studies are needed to validate these observations.

## Figures and Tables

**Table 1:** Radiographic parameters at baseline, post-treatment, and follow-up in children with early-onset scoliosis (n = 15). Normality was assessed using the Shapiro–Wilk test.

Parameter	Time Point	Mean	SD	Min	Max	Normality (Shapiro–Wilk)
Cobb Angle (°)	Pre-treatment	49.5	12.84	26.0	78.0	yes
Cobb Angle (°)	Follow-up	27.33	21.69	8.0	68.0	yes
RVAD (°)	Pre-treatment	29.55	11.64	15.0	50.0	yes
RVAD (°)	Follow-up	14.00	13.49	0.0	47.0	yes
Th1–Th12 Length (cm)	Post-treatment	16.50	1.62	13.4	19.1	yes
Th1–Th12 Length (cm)	Follow-up	19.43	1.45	17.4	21.9	yes
CCW (cm)	Post-treatment	17.03	1.21	15.0	19.2	yes
CCW (cm)	Follow-up	18.15	1.07	16.6	20.0	yes
SAL-L (cm)	Post-treatment	12.50	1.45	9.8	14.7	yes
SAL-L (cm)	Follow-up	15.10	2.05	12.6	18.5	yes
SAL-R (cm)	Post-treatment	11.85	1.49	9.0	13.5	yes
SAL-R (cm)	Follow-up	14.83	2.16	12.3	18.6	yes

Abbreviations: RVAD, rib–vertebra angle difference; CCW, coronal chest width; SAL, space available for lung; SD, standard deviation.

## Data Availability

Data are available upon reasonable request.
